# Porosity, Powder X-Ray Diffraction Patterns, Skeletal Density, and Thermal Stability of NIST Zeolitic Reference Materials RM 8850, RM 8851, and RM 8852

**DOI:** 10.6028/jres.126.047

**Published:** 2022-03-01

**Authors:** Huong Giang T. Nguyen, Ran Tao, Roger D. van Zee

**Affiliations:** 1 Chemical Sciences Division, National Institute of Standards and Technology, Gaithersburg, MD 20899, USA; 2 Materials Measurement Science Division, National Institute of Standards and Technology, Gaithersburg, MD 20899, USA

**Keywords:** characterization, RM 8850, RM 8851, RM 8852, zeolite A, zeolite Y, ZSM-5

## Abstract

This paper reports the powder X-ray diffraction patterns, argon isotherms at 87 K, Brunauer–Emmett–Teller surface areas, pore size distributions, pore volumes, skeletal densities, and thermal gravimetric analyses for three National Institute of Standards and Technology zeolitic reference materials, RM 8850 (zeolite Y), RM 8851 (zeolite A), and RM 8852 (ZSM-5).

## 1 Introduction

Zeolites are porous aluminosilicate minerals that have various industrial applications, such as catalysis, ion exchange, and adsorption [1]. Given the many industrial applications of zeolites, the development of readily available zeolite standards for research intercomparison has received great interest. With support from the National Science Foundation, a 1995 workshop on the need for reference materials in the zeolite community at the California Institute of Technology recommended that the National Institute of Standards and Technology (NIST) develop such standards [2]. NIST undertook work to develop reference materials for three common zeolites, zeolite Y, zeolite A, and ZSM-5 zeolite, which were produced for industrial applications and were donated to NIST. These NIST zeolitic reference materials (RMs), designated as RM 8850 (zeolite Y), RM 8851 (zeolite A), and RM 8852 (ammonium ZSM-5 zeolite) [3-5], are available from NIST at https://www.nist.gov/srm. They are well characterized, with reference values for chemical composition (major components, trace elements, and elemental ratios), loss on ignition (LOI), and loss on fusion (LOF), and informational values for enthalpies of formation, particle size distributions, refractive indices, unit cell parameters, and mass variation with change in relative humidity [2]. For the Report of Investigation, all physicochemical characterization was performed at a near-constant relative humidity (RH) of (54 ± 2) %.

**Fig. 1:**
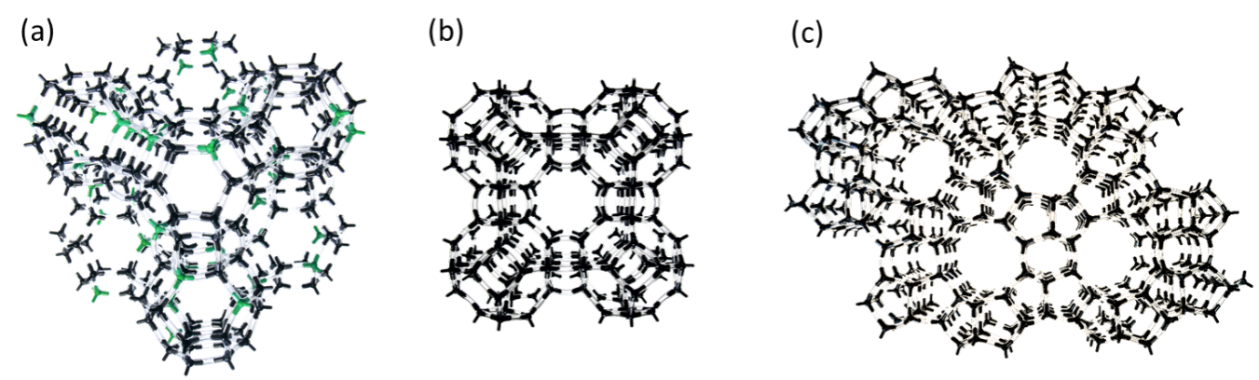
Structures of (a) zeolite Y (RM 8850), (b) zeolite A (RM 8851), and (c) ZSM-5 (RM 8852). Black = silicon or aluminum; green = aluminum; white/clear = oxygen.

RM 8850, or zeolite Y, is a faujasite (FAU) with molecular formula Na_54.0_[Al_54.1_Si_137.9_O_384_]⋅245.1H_2_O [2].^1^1 Turner *et al*. [2] determined the formulas for the zeolites from the reference values of the mass fraction percentages for the major components (Na, Si, Al) and LOI and LOF values.
Zeolite Y was introduced as an acidic zeolitic catalyst for the cracking of hydrocarbons in the 1960s [6]. Its structure is shown in Fig. 1(a). It has a three-dimensional (3D) pore structure in which the pores run in mutually orthogonal directions. The typical pore diameter is defined by an ≈0.8 nm twelve-member oxygen ring that leads into a cavity ≈1.2 nm in diameter. The cavity is surrounded by 10 sodalite cages connected on their hexagonal faces in a tetrahedral 3D structure in which every sodalite cage has four uniformly distributed nearest neighbors as binding partners [6-8].

RM 8851, or zeolite A, is a sodium Linde type A (LTA) zeolite with molecular formula of Na_97.3_[Al_96.2_Si_95.5_O_384_]⋅209.4H_2_O [2]. Zeolite A is the first commercialized synthetic zeolite [9], and it is used widely as a water softener in laundry detergents [10]. The structure of zeolite A is shown in Fig. 1(b); it consists of sodalite cages that are connected by bonds between their four-membered rings to form a 3D network with ≈1.14 nm diameter supercages interconnected by ≈0.41 nm eight-ring openings [11].

RM 8852, or ammonium ZSM-5 (Zeolite Socony Mobil-5), is a Mobil-5 (MFI) zeolite, and it has the molecular formula of (NH_4_)_3.27_[Al_3.27_Si_92.73_O_192_]⋅26.7H_2_O [2]. It is used in catalytic cracking processes. The structure of ZSM-5 is shown in Fig. 1(c). The zeolite consists of chains of eight five-member ring (pentasil) subunits linked to form sheets, which form a 3D framework. The resulting framework has two perpendicular channel systems, with one running straight and the other progressing in a sinusoidal fashion [12].

Recently, two of the NIST zeolitic reference materials were used in NIST-led international interlaboratory studies (ILSs) to address the issue of irreproducibility in adsorption isotherm measurements at high pressures. One study looked at carbon dioxide (CO_2_) adsorption on ammonium ZSM-5 (RM 8852) at 20 °C, and the other looked at methane (CH_4_) adsorption on zeolite Y (RM 8850) at 25 °C [13, 14]. As reference materials, the NIST zeolitic RMs are well characterized and homogenized, making them excellent materials for the interlaboratory studies by eliminating differences in isotherms that could arise from using a material with heterogeneity in chemical or physical properties. This allowed procedural and measurement-focused recommendations to be made [13], and it played a vital part in the resulting high-pressure reference isotherm data from the studies [13, 14]. These studies demonstrated that reproducible isotherms could be obtained with careful sample handling and activation, measurement, and data analysis, and that reference data with small uncertainties could be extracted. These reference adsorption data—available in open-access research articles, at the NIST adsorption database, and as an appendix added to the Report of Investigation of the reference zeolites—should prove useful to evaluate the performance of instruments and the measurement practices of laboratories.

While the reference zeolites have been extensively characterized for chemical composition, the complementary structure and pore characterization of the reference zeolites have not been investigated and reported. In this study, additional types of characterization of these materials were carried out, and the results are reported. The powder X-ray diffraction patterns (PXRD), the argon isotherms at 87 K, the Brunauer–Emmett–Teller (BET) surface areas, the pore size distributions, the pore volume characterizations, the skeletal densities, and the thermal gravimetric analyses of RM 8850, RM 8851, and RM 8852 are provided here. These characterization methods represent those commonly available and utilized in the materials and adsorption communities. They may also further insights into the structure-property relationships of the materials, and they may aid in the development of computational methods looking at adsorption or other measurements [15, 16].

## 2 Experimental Methods and Materials

### 2.1 Materials

The NIST Office of Reference Materials provided the RM 8850, RM 8851, and RM 8852. All samples were used as received without further modifications. Argon (99.999%), helium (99.999%), nitrogen (99.999%), and air were purchased from commercial sources.

### 2.2 Powder X-Ray Diffraction

The PXRD patterns of RM 8850, RM 8851, and RM 8852 were collected on a Bruker D8 Discover X-ray diffractometer (Bruker, Billerica, MA)^2^2 Certain commercial equipment, instruments, or materials are identified in this paper to foster understanding. Such identification does not imply recommendation or endorsement by the National Institute of Standards and Technology, nor does it imply that the materials or equipment identified are necessarily the best available for the purpose. equipped with an EIGER2R 500K detector and a Cu *Kα* radiation X-ray source. The sample powder was packed into the sample holder. The X-ray diffraction pattern scans were collected in the Bragg-Brentano geometry to cover the range of 5° to 50° 2θ in increments of 0.05°. The peaks were integrated, and the diffraction pattern background was subtracted using Bruker DIFFRAC.EVA software (version 5.0).

### 2.3 *Ex Situ* Sample Activation

For measurements made in the helium pycnometer, samples were outgassed *ex situ* in a tube furnace attached to a pumping station that was equipped with a turbomolecular pump (vacuum level of 10^–7^ Pa) backed by a scroll pump (vacuum level of 1 Pa). The following activation protocol was used: under high vacuum (0.1 Pa), the temperature was ramped up from room temperature to 350 °C at a rate of 1 °C/min, held at 350 °C for 12 h, and then cooled (≈7 h) to room temperature to a final vacuum level of 10^–5^ Pa.

After activation, the sample was transferred under air- and moisture-free conditions from the sealed activator tube to an argon glovebox for storage until pycnometry measurements were performed. The mass loss after activation was (6 ± 1) % mass for RM 8852, (25 ± 1) % mass for RM 8850, and (21 ± 1) % mass for RM 8851.

### 2.4 Helium Pycnometer

Skeletal densities were measured using helium as the probe gas in an AccuPyc II 1340 pycnometer (Micromeritics, Norcross, GA). For the pycnometry measurements, the activated sample inside the glovebox was loaded full in one of the pycnometer sample holders. The sample holder was then capped with a fritted cap and quickly transferred (≈5 s) to the pycnometer sample chamber to avoid uptake of moisture and other atmospheric contaminants. Each measurement was made by dosing the sample chamber with helium to ≈134 kPa, which was then allowed to expand into the reference chamber. A sensor determined the pressure to within ± 0.1% uncertainty over the pressure range of vacuum to 207 kPa. The sample volume was determined from the change in pressure and the known volumes of the reference and sample chambers. With the sample volume and sample mass, the skeletal density of the sample could be calculated. The skeletal density of the zeolite presented here is the average value of at least three aliquots. Each measurement collected data points from 50 cycles. The skeletal density of each aliquot was calculated as the average of the last 40 measurement cycles for RM 8852 and the last 30 cycles for RM 8850 and RM 8851, as the equilibrium value was typically reached after 10 to 20 cycles [17]. The expanded uncertainty, *U_k_*_=2_, was taken to be two standard deviations in the skeletal density measurements. The skeletal density of RM 8850 was found to be (2.523 ± 0.014) g/cm^3^, that of RM 8851 was found to be (2.257 ± 0.018) g/cm^3^, and that of RM 8852 was found to be (2.355 ± 0.013) g/cm^3^. The skeletal density value for RM 8852 of (2.349 ± 0.004) g/cm^3^ was previously reported from measurements made at various sample fill volumes [17].

### 2.5 Argon Isotherms

The argon (Ar) adsorption/desorption isotherms up to a pressure of 100 kPa at 87 K were measured in a low-pressure manometric instrument (Autosorb iQ MP, Quantachrome Instruments, Boynton Beach, FL, now a subsidiary of Anton Paar). The temperature was controlled using a liquid argon bath. The sample (approximately 30 mg to 100 mg) was placed inside a 6 mm diameter sample holder that was then loaded with a filler rod to minimize the dead volume. Before each isotherm measurement, the sample was activated on the activation port of the instrument.

The activation procedure was performed under vacuum, ramping from room temperature to 80 °C at a rate of 1 °C/min and holding that temperature for 30 min, then ramping at a rate of 1 °C/min to 120 °C and holding that temperature for 30 min, and finally ramping at a rate of 1 °C/min to 350 °C, where the sample was held for 12 h, before being cooled back down to room temperature.

The BET specific surface area was calculated using the Rouquerol criteria [18] for microporous adsorbents from a BET plot constructed in the appropriate relative pressure (*P*/*P*_0_) range, which in this case was 0.008 ≤ *P*/*P*_0_ ≤ 0.04. Pore size distribution and pore volume were determined from nonlocal density function theory (NLDFT) on the equilibrium branch of the Ar isotherm at 87 K using a kernel for zeolite/silica cylindrical/spherical pores or zeolite/silica cylindrical pores. These calculations were performed using software provided by the manufacturer.

### 2.6 Thermogravimetric Analysis

The thermal decomposition behavior of the reference zeolites was measured using a TA Q500 (TA Instruments, New Castle, DE) thermogravimetric analyzer (TGA) under dry air or nitrogen flow. The mass of TGA samples, placed in 100 μL platinum pans for measurement, ranged from 6 mg to 12 mg. The samples were heated from 30 °C to 800 °C at 10 °C/min.

## 3 Results and Discussion

**Fig. 2:**
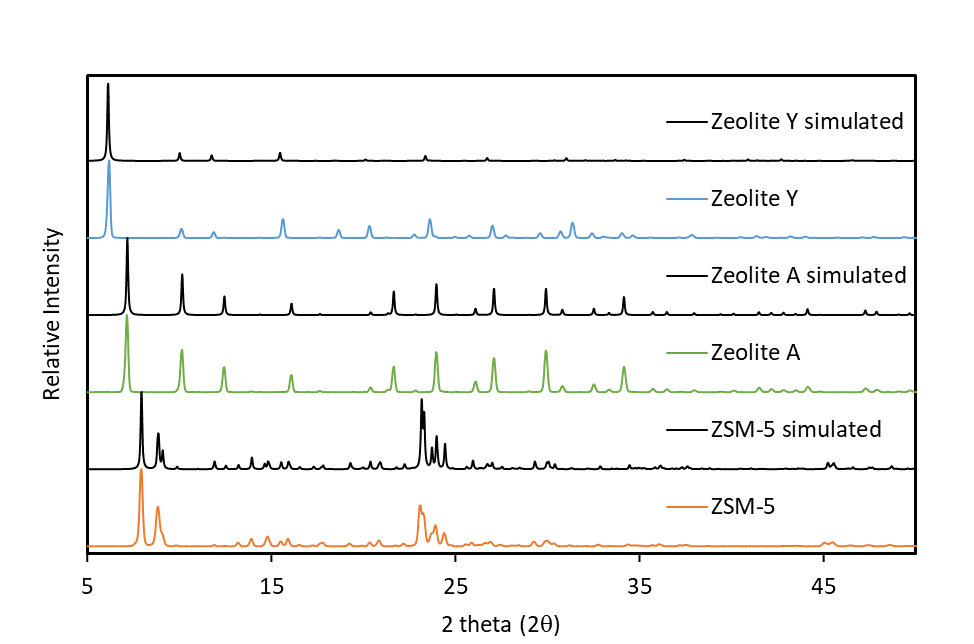
Simulated and experimental powder X-ray diffraction patterns of as-is RM 8850 (FAU), RM 8851 (LTA), and RM 8852 (MFI).

The measured PXRD patterns of RM 8850, RM 8851, and RM 8852 are shown in Fig. 2. The PXRD pattern of each zeolite matches well with its respective simulated pattern, indicating all three zeolites are crystalline. The peaks in the PXRD pattern for as-is (hydrated) zeolite Y (FAU) are slightly shifted from the simulated pattern, which was obtained from the Crystallographic Information File (CIF) of dehydrated zeolite Y by Seo *et al.* [19]. To the best of our knowledge, there is no CIF available for the hydrated form of zeolite Y. The other two simulated patterns were obtained from the CIF files of the hydrated form of LTA [20] and orthorhombic ZSM-5 [21] under MFI zeolite found at http://www.iza-structure.org/databases/. The PXRD pattern of RM 8852 (measured on a different powder diffractometer) has previously been reported and shown to be unchanged before and after exposure to pressure of 4.5 MPa, indicating the material is stable to high pressure; this is further corroborated with the highly reproducible adsorption isotherms over multiple adsorption cycles [22].

The skeletal density measured by helium pycnometry of activated RM 8850 was found to be (2.523 ± 0.014) g/cm^3^, that of activated RM 8851 was found to be (2.257 ± 0.018) g/cm^3^, and that of activated RM 8852 was found to be (2.355 ± 0.013) g/cm^3^. The trend was found to be RM 8850 > RM8852 > RM 8851. All values seem to be reasonable compared to the range of densities observed for SiO_2_ of (2.2 to 2.65) g/cm^3^.

**Fig. 3:**
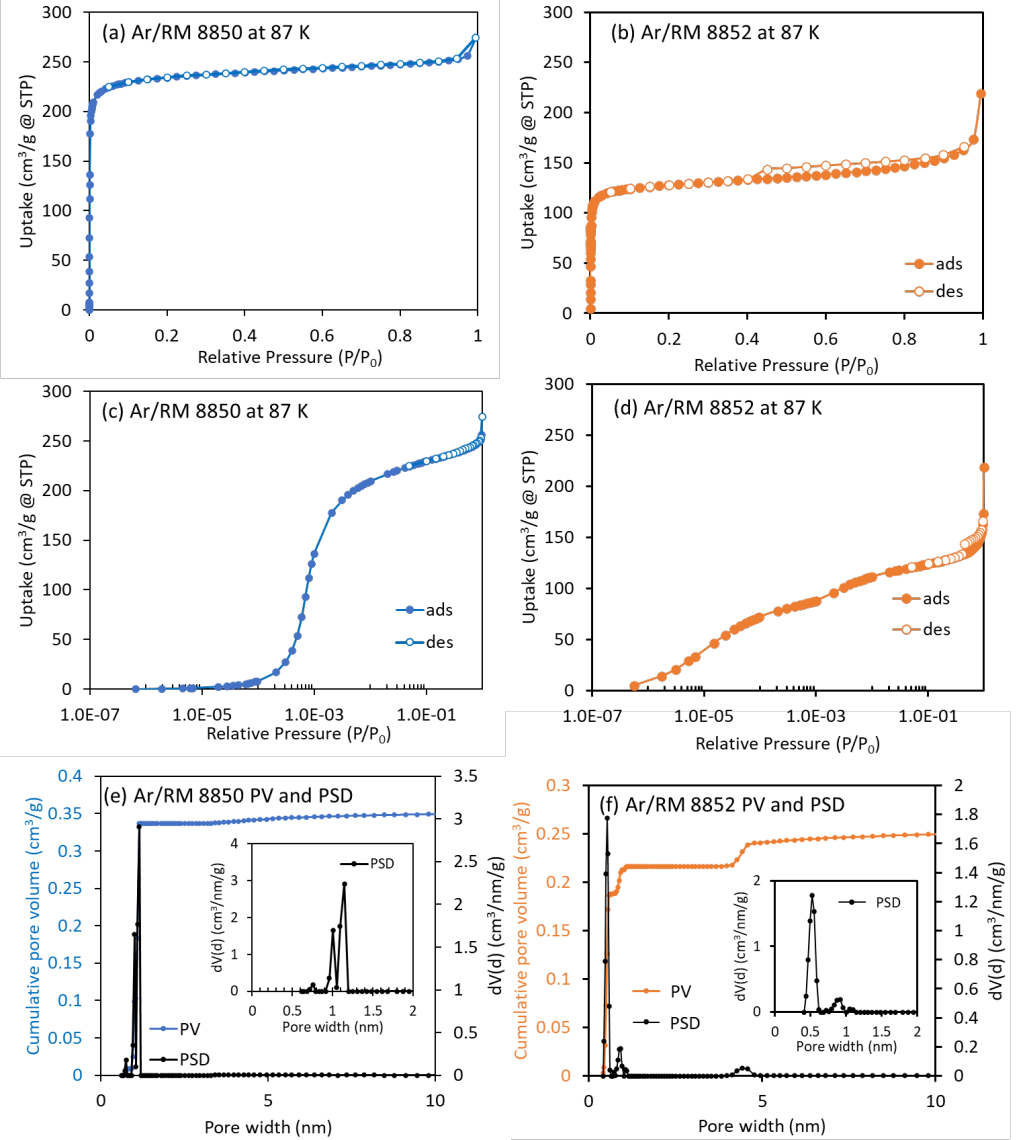
Ar adsorption/desorption isotherms for (a) RM 8850 (FAU) and (b) RM 8852 (MFI); Ar adsorption/desorption isotherms (semilogarithmic scale) for (c) RM 8850 (FAU) and (d) RM 8852 (MFI); and pore size distribution (PSD) and pore volume (PV) for (e) RM 8850 (FAU) and (f) RM 8852 (MFI). STP is standard temperature and pressure.

The Ar isotherms for RM 8850 were previously reported as supporting information in the CH_4_/zeolite Y ILS [14], but no in-depth discussion was provided. The Ar sorption isotherms at 87 K for RM 8850 are shown in Fig. 3(a, c). RM 8850 has a type I isotherm, which is typical for rigid microporous materials. The BET surface area calculated for 0.008 ≤ *P*/*P*_0_ ≤ 0.04 is (819 ± 13) m^2^/g. Figure 3(e) shows the pore size distribution of RM 8850 determined from the NLDFT calculation model on the equilibrium branch of the Ar isotherm at 87 K for zeolites using the spherical/cylindrical pores kernel given that a spherical pores kernel is unavailable. Three peaks were seen at ≈ 0.8 nm and from ≈1.0 nm to ≈1.15 nm in diameter. As the available spherical/cylindrical kernel does not reflect the cage pore structure of zeolite Y, using this kernel gave a NLDFT isotherm with several steps between 1.0 × 10^−4^ ≤ *P*/*P*_0_ ≤ 1.0 × 10^−2^, whereas only one step was observed in the experimental isotherm. The two smaller pore size peaks are likely artifacts from the extra steps in the NLDFT fit, and the main peak (≈1.15 nm in diameter) differs slightly from the expected pore size of ≈1.2 nm for the cages, so it is likely also a result of the imperfect NLDFT fit. The total pore volume of RM 8850 is (0.358 ± 0.003) cm^3^/g, as also determined using the NLDFT calculation model on the equilibrium branch of the Ar isotherm at 87 K for zeolites using the spherical/cylindrical pores kernel.

The Ar sorption isotherms at 87 K for RM 8851 are not available because measurements could not be made after multiple attempts. Given that Ar isotherms at 87 K are preferred over nitrogen (N_2_) isotherms at 77 K for microporous materials, an attempt was not made to measure N_2_ isotherms. Unlike N_2_ at 77 K, Ar at 87 K does not have a quadruple moment, so it is less sensitive to adsorbent surface groups and, thus, can fill micropores at higher relative pressures, leading to accelerated equilibration and permitting the measurement of high-resolution adsorption isotherms [23]. There are currently no Ar sorption isotherms reported for sodium zeolite A in the literature to the best of our knowledge, although high-resolution Ar sorption isotherms have been reported for the calcium version of zeolite A, which is expected to have larger pores [24]. One possible explanation is that the pore windows for sodium zeolite A, which are expected to be ≈0.4 nm, are too small and may be difficult to access or reach equilibrium with Ar.

The Ar sorption isotherms for RM 8852 are shown in Fig. 3(b, d). The Ar sorption isotherms for RM 8852 have a type H4 hysteresis loop above *P*/*P*_0_ = 0.4 (Fig. 3[b]) and a kink in the low-pressure region around *P*/*P*_0_ = 1×10^−3^ (Fig. 3[d]). Isotherms with a type H4 loop have an adsorption branch that is a composite of types I and II, with the pronounced uptake at low *P*/*P*_0_ being attributed to the filling of micropores [23]. High-resolution Ar adsorption isotherms of MFI-type zeolite have been reported to exhibit a kink (coupled with hysteresis) in the low-pressure region [25-27]. This transition has been proposed to be caused by the adsorbate changing from a disordered liquid-like phase to an ordered solid phase [28], or by a monoclinic to orthorhombic phase transition in the adsorbent [29]. Similar structural transition in the zeolite from monoclinic to orthorhombic has been observed with p-xylene [30] and at high temperatures [31, 32]. Garcia-Perez *et al*. supported this hypothesis using molecular dynamic simulations of the Ar isotherm at 77 K, showing that a flexible framework captures the kink/step at the low relative pressure better than a rigid monoclinic or rigid orthorhombic framework [33]. The structure change of MFI-type zeolites was later experimentally confirmed to undergo a monoclinic to orthorhombic structural transition at low relative pressure with Ar at 83 K using *in situ* synchrotron X-ray diffraction [34].

RM 8852 has a BET surface area of (443 ± 5) m^2^/g determined for 0.008 ≤ *P*/*P*_0_ ≤ 0.04. The pore size distribution of RM 8852 determined from the NLDFT calculation model on the equilibrium branch of the Ar isotherm at 87 K for zeolites using the cylindrical pores kernel is shown in Fig. 3(f). RM 8852 consists of pores primarily around ≈0.5 nm in diameter, expected from the channels of ZSM-5, and some mesopores (4.4 nm). The small peak at ≈0.9 nm in diameter is an artifact calculated from the phase transition of ZSM-5 around *P*/*P*_0_ = 1×10^−3^, so it should not exist. The primary NLDFT calculated pore size of RM 8852 of (0.50 ± 0.01) nm is slightly smaller compared to the pore sizes of 0.51 nm to 0.56 nm determined from crystal structure analysis reported for an orthorhombic ZSM-5 (Si/Al = 86) with sodium and tetraalkyl cations [35], and it captures the smaller end of pores sizes of 0.50 nm to 0.58 nm from crystal structure for monoclinic H-ZSM-5 (Si/Al = 299) [36]. While RM 8852 is orthorhombic at room temperature [2], the difference in pore size may be attributed to RM 8852 being monoclinic below *P*/*P*_0_ = 1×10^−3^ for measurements of the Ar isotherms at 87 K. The phase transition around *P*/*P*_0_ = 1×10^−3^ before the micropore filling is completed may also lead to underrepresentation of higher-end pores sizes in the calculation of the pore size distribution. Furthermore, NLDFT is a simplified pore model and may not be able to capture pore wall heterogeneity (*e.g.*, the presence of Al in RM 8852 [Si/Al = 28]) [37], so it is not surprising the NLDFT-predicted pore size distribution does not exactly match that from crystal structure analysis. The total pore volume of RM 8852 is (0.272± 0.005) cm^3^/g as determined by NLDFT calculations. Modeling work relies on pore characterization data to perform accurate simulation. Modeling by Fang *et al*. showed good agreement between the simulated isotherm and the reference CO_2_/ZSM-5 isotherm at 20 °C after taking into account the small amount of mesoporosity and deammoniation of the ammonium ions during pretreatment [38].

**Fig. 4:**
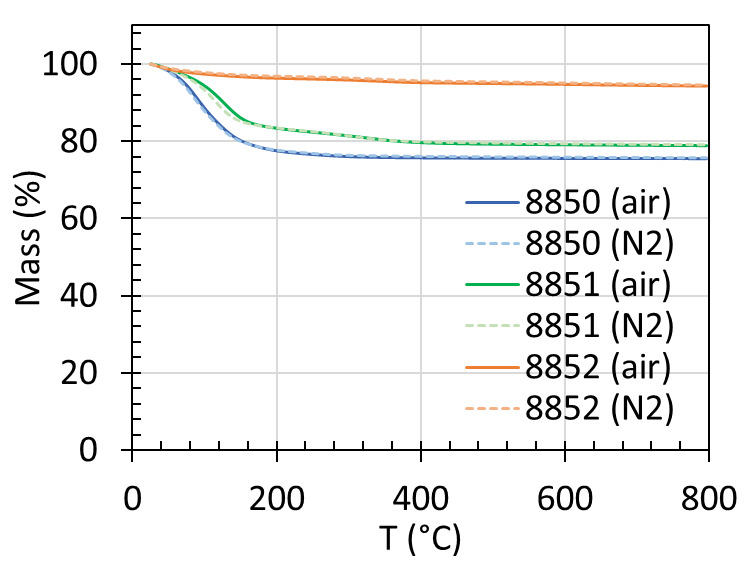
Thermal gravimetric analysis of RM 8850, RM 8851, and RM 8852 in air (continuous line) and in nitrogen (dotted line).

The TGA plots of mass (%) as a function of temperature for the three as-is NIST reference zeolites are shown in Fig. 4. The TGA profiles show a noticeable mass loss starting around 90 °C to 100 °C for RM 8850 and RM 8851, which is attributed to water. A smaller, more gradual mass loss is observed in RM 8852. All three zeolites are thermally stable in air and in nitrogen up to 800 °C, the maximum temperature of the reported TGA measurements. The mass loss at 800 °C is 24.3%, 20.9%, and 5.4% for RM 8850, RM 8851, and RM 8852, respectively. The mass loss values agree well (within the uncertainty) with values typically observed after activating the samples at 350 °C for 12 h under high vacuum. The mass loss after activation for RM 8850 was (25 ± 1) % mass, that for RM 8851 was (21 ± 1) % mass, and that for RM 8852 was (6 ± 1) % mass, suggesting the activation condition was sufficient to remove all physiosorbed species. These values are also consistent with theoretical values for the mass of water calculated from the

molecular formula of the hydrated (at 54% RH) forms of the zeolites (see Sec. 1): 23.3%, 19.6%, and 6.7% mass water for RM 8850, RM 8851, and RM 8852, respectively. In the case of RM 8852, deammoniation may occur during activation [39], and the theoretical mass value for water and the ammonium ions is 7.7%. The measured mass loss values are also similar to previously reported LOI values of (25.679 ± 0.095) %, (21.464 ± 0.085) %, and (8.50 ± 0.09) %, and LOF values of (25.37 ± 0.67) %, (22.1 ± 1.7) %, and (8.47 ± 0.38) % for RM 8850, RM 8851, and RM 8852, respectively [2]. The small observed difference, especially for RM 8852, may be due to samples in this work being studied as-is without subjecting them to 54% RH. While the formulas at 54% RH for RM 8850 and RM 8851 reported by Turner *et al*. [2] contained approximately similar numbers of water molecules as generic formulas for zeolite Y and zeolite A, respectively, there are significantly more water molecules (26.7 vs. 16) reported for RM 8852 at 54% RH compared to the generic formula for ZSM-5. Study of mass change *vs*. humidity for RM 8852 indicated its mass change is more sensitive to % RH (with up to 1.3% mass change from 32% to 54% RH) than RM 8850 and RM 8851 [3-5]. The theoretical mass value calculated for water is 4.1% mass, and that for water and ammonia is 5.1% mass using the water content from the generic formula for ZSM-5. Modeling by Fang *et al*. [38] has suggested deammoniation to take place in RM 8852 during activation, showing good agreement between the simulated isotherm and the reference CO_2_/ZSM-5 isotherm at 20 °C after taking into account the small amount of mesoporosity and deammoniation of the ammonium ions during pretreatment.

## 4 Conclusions

This article reports additional characterization of the NIST zeolitic reference materials RM 8850, RM 8851, and RM 8852. In particular, the powder X-ray diffraction patterns, Ar isotherms at 87 K, surface areas, pore size distributions, pore volumes, skeletal densities, and thermal gravimetric analysis profiles of NIST RM 8850, RM 8851, and RM 8852 are reported. Along with the Reports of Investigation, and previous reports, these data should be helpful to laboratories interested in using the NIST zeolitic reference materials.
